# Association Between Anterior Maxillary Sinus Wall Position and Nasal Bone Asymmetry with Patient-Reported Outcomes Following Septorhinoplasty

**DOI:** 10.3390/jcm15093250

**Published:** 2026-04-24

**Authors:** Meysem Yorgun, Erdinc Cekic, Adem Topcu, Recep Haydar Koc, Ozgur Surmelioglu

**Affiliations:** 1 Otolaryngology Department, MMT American Hospital, Reyhanlı, 31500 Hatay, Turkey; meysemyorgun@gmail.com; 2 Department of Otolaryngology, Haseki Education and Research Hospital, Health Science University, Fatih, 34096 Istanbul, Turkey; erdinccekic@gmail.com (E.C.); recephaydarkoc@gmail.com (R.H.K.); 3 Department of Radiology, Haseki Education and Research Hospital, Health Science University, Fatih, 34096 Istanbul, Turkey; ademtopci@gmail.com; 4 Department of Otorhinolaryngology, Faculty of Medicine, Çukurova University, Sarıçam, 01330 Adana, Turkey

**Keywords:** septorhinoplasty, nasal bone asymmetry, maxillary sinus anatomy, patient satisfaction, nasal septum deviation

## Abstract

**Objectives**: To evaluate the relationship between anterior maxillary sinus wall position and nasal bone asymmetry, and to assess their association with patient-reported aesthetic and functional outcomes following septorhinoplasty. **Methods**: Preoperative and postoperative evaluations were performed using the Rhinoplasty Outcome Evaluation (ROE) and Nasal Obstruction Symptom Evaluation (NOSE) questionnaires. Nasal bone dimensions were measured preoperatively using computed tomography (CT) scans. **Results**: A positive correlation was observed between anterior maxillary sinus wall position and nasal bone asymmetry, with significant nasal bone discrepancies contributing to nasal deviation. **Conclusions**: Anterior maxillary sinus wall position and nasal bone asymmetry are significantly associated with patient-reported outcomes following septorhinoplasty. Consideration of these anatomical parameters during surgical planning may improve aesthetic and functional results.

## 1. Introduction

Facial asymmetry, defined by variations in the alignment and structure of the facial bones, significantly affects both aesthetics and function. The maxillary position and nasal bone structure are critical factors influencing overall facial harmony. Anatomical variations in the maxillary region, particularly the anterior maxillary sinus wall position, may influence nasal skeletal configuration and overall nasal contour [1]. In addition, maxillary anatomical configuration may also affect airway-related outcomes and should therefore be considered during preoperative septorhinoplasty planning [2].

Nasal bone discrepancy is another vital consideration for facial asymmetry correction. Differences in the nasal bone structure can lead to noticeable facial imbalances even when the maxillary position is corrected. Addressing nasal bone asymmetry during septorhinoplasty may enhance aesthetic balance, contributing to a more symmetrical facial profile and improved patient satisfaction [3,4]. This highlights the importance of considering both maxillary anatomical variation and nasal bone asymmetry during preoperative septorhinoplasty planning [5].

Patient satisfaction is a crucial measure of success in facial reconstructive procedures. Studies have consistently shown that patients who perceive a significant improvement in facial symmetry and balance report higher satisfaction levels postoperatively [6,7]. This underscores the importance of considering both functional and aesthetic outcomes in surgical planning. Moreover, advancements in surgical techniques and preoperative planning have improved the predictability of outcomes, further enhancing patient satisfaction and quality of life following septorhinoplasty and related nasal corrective procedures [8,9].

## 2. Material and Methods

### 2.1. Study Environment and Participants

This study was conducted at the University of Health Sciences, Tertiary Training and Research Hospital. A total of 79 patients, who presented to the Otolaryngology (ENT) Clinic of our hospital with complaints of nasal septum deviation and were scheduled for septorhinoplasty surgery, were included in the study. The patients consisted of individuals who underwent Paranasal Sinus Computed Tomography (CT) performed by the Radiology Department, septorhinoplasty surgery performed by single surgeon, and who had a follow-up period of at least 6 months.

### 2.2. Population/Sample of the Study

The population of the study consists of patients who presented to the ENT Clinic of our hospital with complaints of nasal septum deviation, were scheduled for septorhinoplasty surgery, underwent Paranasal Sinus CT performed by the Radiology Department, had septorhinoplasty surgery performed by single surgeon, had at least 6 months of follow-up, and agreed to participate in the study.

### 2.3. Ethical Approval and Informed Consent

Ethical approval for the study was obtained from the Clinical Research Ethics Committee of the University of Health Sciences Tertiary Training and Research Hospital (date: 29 August 2024, decision no: 67-2024). Informed consent forms were obtained from all patients to ensure their participation in the study. Throughout the study, utmost attention was paid to patient confidentiality and ethical standards.

In surgical planning, nasal bone asymmetry and anterior maxillary sinus wall position were taken into consideration when determining the extent of bony modification.

### 2.4. Data Collection Method

All patients with planned septorhinoplasty and nasal septum deviation underwent routine Paranasal Sinus Computed Tomography (CT). Rhinoplasty outcome evaluation (ROE) and Nasal Obstruction Symptom Evaluation (NOSE) questionnaires were administered to all participating patients during both the preoperative and postoperative periods. These questionnaires were completed preoperatively and at a standardized postoperative time point of 6 months. The ROE scale ranges from 0 to 24, with higher scores indicating greater patient satisfaction, while the NOSE scale ranges from 0 to 20, with higher scores indicating more severe nasal obstruction symptoms. The ROE questionnaire assesses the patients’ satisfaction in terms of aesthetics and functionality, while the NOSE questionnaire measures the severity of nasal obstruction symptoms.

**Figure 1:**
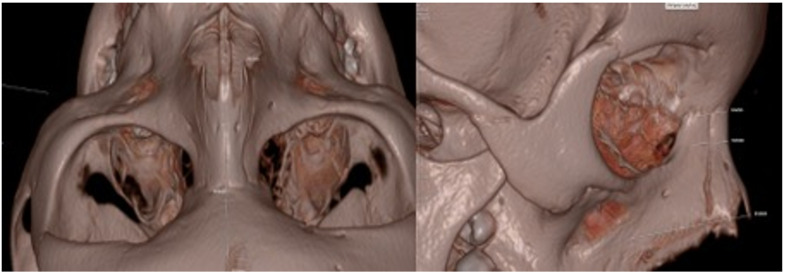
The Longitudinal Length of The Right/Left Nasal Bone and R1-R2-R3 measurements.

**Figure 2:**
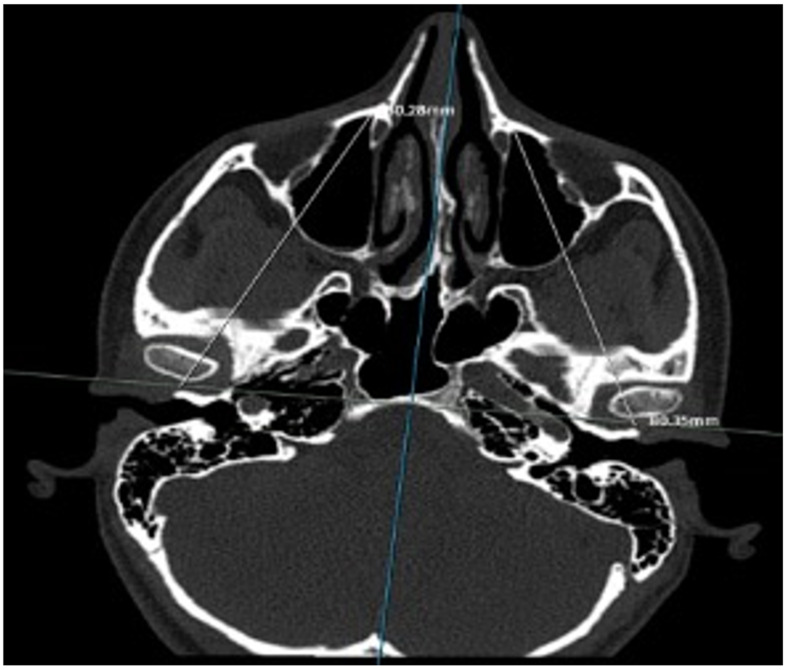
The Distance from the Temporomandibular Joint to The Anterior Wall of the Maxillary Sinus.

Preoperative CT scans were examined by experienced radiologists, and nasal bone dimensions were measured at four points on both the right and left sides. During these measurements, the lengths of the right and left nasal bone walls were determined, and differences between both sides were noted. The anterior walls of the maxillary sinuses were evaluated based on a horizontal line passing through both temporomandibular joints. A total of 10 different data points were obtained for each patient from these measurements. The relationships between the nasal bone sizes on the right and left sides, the differences between the two sides, and the maxillary sinus anterior walls were examined. The impact of these differences on patient satisfaction was also evaluated (Figure 1 and Figure 2).

### 2.5. Surgical Intervention

All patients underwent surgery under general anesthesia by an experienced surgical team following standard procedures. The surgical field was isolated by using sterile drapes. After the injection of jetocaine, a transcolumellar ‘W’ incision was made, the dorsal flap was elevated, and supraperichondrial and subperiosteal elevations were completed. Following removal of the dorsal hump, the nasal bones were measured, and considering the anterior positioning of the maxillary sinus, the nasal bone was left 1–2 mm shorter on the anterior and medial sides. Bone resections and cuts were performed using a piezoelectric device (Mectron Medical Technology, Carasco, Italy), which allowed more controlled and precise cutting. Calipers were used to measure the nasal bone and maxillary structures. Advances in surgical techniques and patient-centered approaches have been considered, and the most appropriate surgical intervention has been applied to each patient. Transverse and lateral osteotomies were performed.

### 2.6. Statistical Analysis

All statistical analyses were conducted using IBM SPSS Statistics for Windows, Version 27.0 (IBM Corp., Armonk, NY, USA). Continuous variables were expressed as mean ± standard deviation (SD). The normality of the data distribution was assessed using the Shapiro–Wilk test. For normally distributed variables, paired or unpaired *t*-tests were used to compare preoperative and postoperative measurements, where appropriate. Pearson correlation analysis was performed to evaluate the relationships between maxillary sinus measurements and nasal bone parameters (R1, R2, R3, MR, L1, L2, L3, and ML). Additional correlation analyses were performed to assess the association between anatomical asymmetry parameters and changes in ROE and NOSE scores. Variables showing significant associations were subsequently entered into a linear regression model to identify independent predictors of postoperative improvement in ROE scores. A *p*-value of less than 0.05 was considered statistically significant.

The sample size was calculated as the ratio of the difference between the mean and the standard deviation of both groups. The G * Power 3.1 program (Düsseldorf, Germany) was used for post hoc power analysis. The alpha significance level was 0.05, and the 95% power was calculated as 78, assuming an effect size difference of 0.8. According to this result, power analysis indicated a minimum sample size of 78.

## 3. Results

A total of 79 patients were included in the study. Ethical approval was obtained from the Ethics Committee of Haseki Education and Research Hospital (Decision No:67-2024, Date: 29 August 2024). Rhinoplasty was performed on 35 male (44.3%) and 44 female (55.7%) patients.

The study cohort had a mean age of 26.9 years (±6.7). The mean Body Mass Index (BMI) was 21.5 kg/m^2^ (±2.4). Maxillary measurements showed that the mean right maxillary length was 84.04 mm (±4.94), while the mean left maxillary length was 82.78 mm (±4.88). Specific regional measurements revealed that the mean R1 value was 12.1 mm (±1.6), R2 was 12.7 mm (±1.6), and R3 was 32.1 mm (±4.2). Corresponding measurements on the left side indicated that L1 was 11.3 mm (±1.6), L2 was 12.3 mm (±1.6), and L3 was 31.7 mm (±3.4). Additionally, the mean MR measurement was 27.6 mm (±3.2), and the mean ML measurement was 27.8 mm (±3.0) (Table 1).

The mean preoperative Rhinoplasty Outcome Evaluation (ROE) score had a mean value of 3.8 (±2.3), which significantly increased to 16.9 (±2.8) postoperatively. Similarly, the preoperative Nasal Obstruction Symptom Evaluation (NOSE) score had a mean value of 11.8 (±5.5) and showed a considerable decrease to 3.5 (±3.4) following surgery. The average follow-up duration for the patients was 14.8 months (±6.0) (Table 2).

The correlation analysis between the right maxillary sinus measurements and the R1, R2, R3, and MR parameters revealed the following results: The correlation coefficient for R1 was 0.310 (*p* = 0.005), for R2 it was 0.304 (*p* = 0.006), for R3 it was 0.220 (*p* = 0.051), and for MR it was 0.129 (*p* = 0.257) (Table 3).

Correlation analysis between the left maxillary sinus measurements and the L1, L2, L3, and ML parameters demonstrated the following results: the correlation coefficient for L1 was 0.262 (*p* = 0.020), for L2 it was 0.223 (*p* = 0.048), for L3 it was 0.177 (*p* = 0.119), and for ML it was 0.319 (*p* = 0.004) (Table 4).

A significant positive correlation was observed between nasal bone asymmetry and improvement in ROE scores (r = 0.32, *p* = 0.006), while a negative correlation was found with ΔNOSE scores, indicating greater functional improvement in patients with higher preoperative asymmetry (Table 5).

Linear regression analysis demonstrated that nasal bone asymmetry (β = 0.35, *p* = 0.004) and maxillary asymmetry (β = 0.29, *p* = 0.011) were independent predictors of postoperative improvement in ROE scores (Table 6).

## 4. Discussion

This study highlights a clinically relevant association between anterior maxillary sinus wall position and nasal bone asymmetry in patients undergoing septorhinoplasty. Our findings suggest that variation in anterior maxillary sinus wall position is associated with differences in nasal skeletal configuration, which may be relevant in preoperative planning. These results support consideration of maxillary sinus anatomy during planning of septorhinoplasty and related bony correction. Importantly, our additional analyses demonstrated that both nasal bone asymmetry and maxillary anatomical variation were independently associated with improvements in patient-reported outcomes. These findings strengthen the clinical relevance of preoperative anatomical assessment and suggest that individualized surgical planning based on maxillary sinus anatomy may enhance postoperative satisfaction.

The relationship between nasal septum deviation and maxillary sinus anatomy has been explored in various studies, which typically focus on sinus volume rather than elongation. Kalsotra et al. conducted a prospective observational study that examined the impact of nasal septum deviation severity on maxillary sinus volume and the occurrence of sinusitis [10]. They found that, as the grade of nasal septum deviation increased, there was a corresponding decrease in maxillary sinus volume on the side of the deviation, along with an increased incidence of sinusitis due to osteomeatal complex (OMC) blockage. This study provides a strong basis for our findings, as it highlights the direct impact of septal deviation on sinus volume and function, reinforcing the need to consider these anatomical variations in surgical planning

The relationship between nasal septum deviation and maxillary sinus anatomy has been a subject of interest in the literature, although most studies have focused on sinus volume rather than on elongation. Rodriguez Betancourt et al. investigated the relationship between nasal septum deviation and maxillary sinus volume using Cone Beam Computed Tomography (CBCT), but found no significant correlation between the deviation and decreased maxillary sinus volume. However, their study did not consider the impact of sinus elongation, which has been identified as a potentially critical factor influencing septal deviation [11].

Our findings align with previous research that underscores the importance of adjacent sinus structures in nasal surgery. For instance, Sapmaz et al. demonstrated that angulation of the hard palate caused by septal deviation could affect the maxillary sinus volume, which in turn might influence the outcomes of septoplasty. This study emphasizes the complexity of sinus anatomy and its potential impact on nasal surgery, reinforcing the need to consider anterior maxillary sinus wall position during preoperative planning [12]. Moreover, the influence of maxillary sinus anatomy on the nasal structure has broader clinical implications. Lawson et al. explored the development and pathologic processes affecting maxillary sinus pneumatization, highlighting the dynamic nature of the sinus anatomy and its significant role in facial growth and nasal function. This is in line with our view that anterior maxillary sinus wall position may be relevant during preoperative assessment and surgical planning, particularly with regard to optimizing postoperative aesthetic and functional outcomes [13].

The importance of detailed preoperative assessment is further supported by Orhan et al., who conducted a morphometric analysis of the maxillary sinus in patients with nasal septal deviation. They found that variations in sinus anatomy could significantly impact nasal airway function, underscoring the need for a tailored approach to nasal surgery. Our study builds on these findings by suggesting that anterior maxillary sinus wall position, rather than just volume, plays a crucial role in the dynamics of nasal septal deviation [14].

Previous studies have emphasized that craniofacial morphology represents a complex, multidimensional system that cannot be fully characterized using limited regional measurements alone. Comprehensive analyses of facial asymmetry typically rely on three-dimensional landmark-based approaches and volumetric assessments across multiple craniofacial compartments, allowing evaluation of both global and regional morphological interactions [15]. Similarly, studies investigating the relationship between nasal septum deviation and nasal bone morphology have demonstrated that localized anatomical variations may influence adjacent structures without necessarily reflecting a broader craniofacial asymmetry pattern [16]. In line with these findings, our results should be interpreted within the context of localized anatomical relationships rather than global facial asymmetry. While we identified a significant association between anterior maxillary sinus wall position and nasal bone asymmetry, our measurement approach was intentionally focused on specific nasomaxillary parameters rather than comprehensive craniofacial metrics. Previous morphometric studies have shown that the interaction between the nasal cavity and maxillary sinus is highly dependent on regional anatomical configuration, particularly nasal cavity shape and internal dimensions, rather than overall craniofacial symmetry [17]. Therefore, our findings contribute to the existing literature by demonstrating that localized variations in maxillary sinus anatomy are associated with nasal bone asymmetry and patient-reported outcomes in septorhinoplasty patients, without implying a direct representation of global facial asymmetry. This targeted approach may be particularly relevant for surgical planning, where localized anatomical relationships often have greater clinical importance than global craniofacial metrics.

Despite the valuable insights provided by this study, several limitations of this study must be acknowledged. The sample size, which was sufficient to detect significant correlations, may not be representative of the broader population. Additionally, this study was conducted at a single institution, which could introduce selection bias. Although 2D CT imaging was utilized due to its routine clinical availability and standardization, it may not fully represent the complex three-dimensional anatomical relationships of the maxillary sinus and nasal structures. Future studies incorporating 3D imaging modalities such as CBCT or 3D reconstruction are warranted to validate and extend these findings. It should be emphasized that the parameters used in this study represent localized anatomical features rather than a comprehensive assessment of global facial asymmetry. Future research should incorporate larger and more diverse samples and advanced imaging modalities to validate and extend these findings. Long-term follow-up studies would also be beneficial in assessing the persistence of functional and aesthetic improvements associated with adjusting osteotomy techniques based on anterior maxillary sinus wall position.

Preoperative CT scans are essential for understanding nasal bone anatomy and for effective preoperative planning. Our study found that the ipsilateral nasal bones were larger in patients with an elongated anterior maxillary sinus wall, demonstrating a statistically significant positive correlation. This finding may help explain deviation of the nose toward the contralateral side and suggests a possible mechanism contributing to crooked nose deformity in non-traumatic cases. Intraoperative assessment of nasal bone dimensions may support more individualized bony modification and may contribute to improved postoperative outcomes. During the healing period following rhinoplasty, new bone formation occurs in the osteotomized area between the maxilla and nasal bones, re-establishing the connection within months. Due to the anterior positioning of the maxillary wall, this connection may occur at a greater angle, potentially causing the nose to deviate back towards the opposite side, leading to recurrence of the deformity months later. Therefore, we hypothesize that leaving the ipsilateral nasal bone 1–2 mm shorter may reduce the risk of postoperative deviation recurrence; however, this assumption requires validation through long-term prospective imaging studies.

## Figures and Tables

**Table 1:** Demographic and Maxillary Measurements of the Study Cohort.

	Mean ± S.D.
**Age (years)**	26.9 ± 6.7
**BMI (kg/m^2^)**	21.5 ± 2.4
**Right Maxillary (mm)**	84.04 ± 4.94
**Left Maxillary (mm)**	82.78 ± 4.88
**R1 (mm)**	12.1 ± 1.6
**R2 (mm)**	12.7 ± 1.6
**R3 (mm)**	32.1 ± 4.2
**L1 (mm)**	11.3 ± 1.6
**L2 (mm)**	12.3 ± 1.6
**L3 (mm)**	31.7 ± 3.4
**MR (mm)**	27.6 ± 3.2
**ML (mm)**	27.8 ± 3.0

**Table 2:** Pre- and Postoperative Evaluation of ROE and NOSE Scores with Follow-Up Duration.

	Mean ± S.D.
**ROE—Pre**	3.8 ± 2.3
**ROE—Post**	16.9 ± 2.8
**NOSE—Pre**	11.8 ± 5.5
**NOSE—Post**	3.5 ± 3.4
**Follow-up (month)**	14.8 ± 6.0

**Table 3:** Correlation Between Right Maxillary Sinus Measurements and R1, R2, R3, and MR Parameters.

		R1	R2	R3	MR
**Right Maxillary Sinus**	*Correlation*	0.310	0.304	0.220	0.129
	*p value*	**0.005**	**0.006**	0.051	0.257
	*N*	79	79	79	79

**Table 4:** Correlation Between Left Maxillary Sinus Measurements and L1, L2, L3, and ML Parameters.

		L1	L2	L3	ML
**Left Maxillary Sinus**	*Correlation*	0.262	0.223	0.177	0.319
	*p value*	0.020	0.048	0.119	0.004
	*N*	79	79	79	79

**Table 5:** Correlation Between Anatomical Parameters and Changes in ROE and NOSE Scores.

Parameter	ΔROE (r)	*p*-Value	ΔNOSE (r)	*p*-Value
Nasal bone asymmetry	R–L	mean	0.32	**0.006**
Maxillary asymmetry (Right–Left difference)	0.27	0.018	−0.22	**0.041**
MR–ML difference	0.30	0.009	−0.25	**0.021**

**Table 6:** Linear Regression Analysis Predicting Postoperative Improvement in ROE Scores.

Variable	β	Standard Error	*p*-Value
**Nasal bone asymmetry**	0.35	0.12	**0.004**
**Maxillary asymmetry**	0.29	0.11	**0.011**
**Age**	0.08	0.07	0.312
**Gender**	0.05	0.09	0.544

## Data Availability

The datasets generated and/or analyzed data during the current study are available from the corresponding author upon reasonable request.

## References

[1] Avrămuț R.P., Stăncioiu A.A., Talpos S., Motofelea A.C., Popa M., Szuhanek C. (2025). Quantitative Evaluation of Skeletal, Dental, and Soft Tissue Changes After Orthognathic Surgery: A Cephalometric and Statistical Analysis. J. Clin. Med..

[2] Udomlarptham N., Lin C.-H., Wang Y.-C., Ko E.-C. (2018). Does two-dimensional vs. three-dimensional surgical simulation produce better surgical outcomes among patients with class III facial asymmetry?. Int. J. Oral Maxillofac. Surg..

[3] Jadczak M., Krzywdzińska S., Rozbicki P., Jurkiewicz D. (2024). The Crooked Nose-Surgical Algorithm in Post-Traumatic Patient-Evaluation of Surgical Sequence. J. Clin. Med..

[4] Scott B.L., Pearlman S. (2024). Nasal Deviation and Facial Asymmetry in Patients Undergoing Rhinoplasty. Aesthet. Surg. J..

[5] Johnsen S.G. (2024). Computational Rhinology: Unraveling Discrepancies between In Silico and In Vivo Nasal Airflow Assessments for Enhanced Clinical Decision Support. Bioengineering.

[6] Kim Y.J., Kim M.Y., Jha N., Jung M.H., Kwon Y.D., Shin H.G., Ko M.J., Jun S.H. (2024). Treatment outcome and long-term stability of orthognathic surgery for facial asymmetry: A systematic review and meta-analysis. Korean J. Orthod..

[7] Uppada U.K., Tauro D., Senthilnathan K.P. (2023). Patient Satisfaction Following Orthognathic Surgery: A Systematic Review. J. Maxillofac. Oral. Surg..

[8] Thiem D.G.E., Schneider D., Hammel M., Saka B., Frerich B., Al-Nawas B., Kämmerer P.W. (2021). Complications or rather side effects? Quantification of patient satisfaction and complications after orthognathic surgery-a retrospective, cross-sectional long-term analysis. Clin. Oral Investig..

[9] Cheng L.H., Roles D., Telfer M.R. (1998). Orthognathic surgery: The patients’ perspective. Br. J. Oral Maxillofac. Surg..

[10] Kalsotra G., Saroch P., Gupta A., Kalsotra P., Saraf A. (2023). The Variations in Deviation of Nasal Septum and their Impact on Maxillary Sinus Volume and Occurrence of Sinusitis. Indian J. Otolaryngol. Head Neck Surg..

[11] Rodriguez Betancourt A.B., Martinez Somoza L.J., Romero Mesa C., Tozum T.F., Mourão C.F., Shibli J.A., Suárez L.J. (2024). Relationship of Maxillary Sinus Volume and Nasal Septum Deviation: A Cone Beam Computed Tomography Study. Diagnostics.

[12] Sapmaz E., Kavaklı A., Sapmaz H.I., Ögetürk M. (2018). Impact of Hard Palate Angulation Caused by Septal Deviation on Maxillary Sinus Volume. Turk. Arch. Otorhinolaryngol..

[13] Lawson W., Patel Z.M., Lin F.Y. (2008). The development and pathologic processes that influence maxillary sinus pneumatization. Anat. Rec..

[14] Orhan I., Ormeci T., Aydin S., Altin G., Urger E., Soylu E., Yilmaz F. (2014). Morphometric analysis of the maxillary sinus in patients with nasal septum deviation. Eur. Arch. Otorhinolaryngol..

[15] Liang C., Profico A., Buzi C., Khonsari R.H., Johnson D., O’Higgins P., Moazen M. (2023). Normal human craniofacial growth and development from 0 to 4 years. Sci. Rep..

[16] Serifoglu I., Oz İ.İ., Damar M., Buyukuysal M.C., Tosun A., Tokgöz Ö. (2017). Relationship between the degree and direction of nasal septum deviation and nasal bone morphology. Head Face Med..

[17] Holton N., Yokley T., Butaric L. (2013). The morphological interaction between the nasal cavity and maxillary sinuses in living humans. Anat. Rec..

